# Supplementary material for: Principal cells of the brainstem’s interaural sound level detector are temporal differentiators rather than integrators
Source: eLife. 2018 Jun 14;7:e33854. doi: 10.7554/eLife.33854 (PMC6063729; doi:10.7554/eLife.33854)
Supplement: Supplementary file 1. — Overview of morphological and physiological properties of labeled LSO neurons. [file elife-33854-supp1.docx]

**Supplementary Table**

| **Cell number** | **Morphological cell type** | **CF (BF) (kHz)** | **Synaptic coverage (%)** | **IC proj.** | **Axon  collaterals** | **AP amplitude (mV)** | **PSTH** | **IR p/ss (MΩ)** |
| --- | --- | --- | --- | --- | --- | --- | --- | --- |
| **1** | P | 3.5 | 68.0 | I |  | 22.1 | O | 13/2 |
| **2** | P | 4.1 | 74.8 | I | LNTB | 48.0 | - |  |
| **3** | P | (4.6) | 59.1 |  |  | 17.9 | - |  |
| **4** | P | 7.5 | 77.4 |  |  | 25.9 | O |  |
| **5** | P | 12.0 | 76.4 | I | LNTB | 27.6 | O | 28/18 |
| **6** | P or 5 | 3.8 |  |  |  | 19.2 | O(s) |  |
| **7** | P or 5 | 5.7 |  |  |  | 36.2 | O |  |
| **8** | P or 5 | 10.4 |  |  |  |  | (O) |  |
| **9** | P or 5 | (0.29) |  |  |  |  | - |  |
| **10** | Ma | 4.1 | 53.8 |  |  | 48.9 | Chop (T) |  |
| **11** | Ma | 2.3 |  |  |  | 65.5 | O |  |
| **12** | Ma | 4.0 | 42.9 | I |  | 56.0 | PL |  |
| **13** | Ma | (10.6) | 52.4 | I | local | 80.1 | PL | 37/28 |
| **14** | Ma | 5.7 | 27.8 | C |  | 60.1 | Chop (U) | 59/48 |
| **15** | Mu | 4.7 | 75.3 | I | LNTB; local | 72.4 | O(s) | 44/19 |
| **16** | 5 | 2.0 | 32.8 | bilat. | local | 79.7 | O(s) |  |

**Supplementary Table.** Overview of morphological and physiological properties of labeled LSO neurons. Cell type - P: principal cell; 5: Type 5; Ma: marginal cell; Mu: multiplanar cell. CF - number in parentheses indicate BF (all at 60 dB). IC projection - I: ipsilateral; C: contralateral. PSTH (peristimulus time histogram) - O: onset; O(s): onset followed by low level sustained activity; chop: chopper (type T or U); PL: primary-like. IR (input resistance - peak/steady state). For cell 8, which was morphologically compatible with either a principal cell or a non-principal (class 5) neuron, we did not obtain a reliable measure for action potential amplitude because it was heavily depolarized when it started firing. We did not obtain a full PSTH for this neuron, but the responses obtained during frequency tuning showed firing at sound onset. For three other labeled cells, we did not obtain enough data to be able to evaluate the PSTH to tones at CF or BF.
