# Supplementary material for: Principal cells of the brainstem’s interaural sound level detector are temporal differentiators rather than integrators
Source: eLife. 2018 Jun 14;7:e33854. doi: 10.7554/eLife.33854 (PMC6063729; doi:10.7554/eLife.33854)
Supplement: Transparent reporting form [file elife-33854-transrepform.pdf]

eLIFE
1st Floor
24 Hills Road
Cambridge CB2 1JP, UK
P 01223 855340
W elifesciences.org
T @elife

eLife's transparent reporting form

We encourage authors to provide detailed information *within their submission* to facilitate the interpretation and replication of experiments. Authors can upload supporting documentation to indicate the use of appropriate reporting guidelines for health-related research (see EQUATOR Network), life science research (see the BioSharing Information Resource), or the ARRIVE guidelines for reporting work involving animal research. Where applicable, authors should refer to any relevant reporting standards documents in this form.

If you have any questions, please consult our Journal Policies and/or contact us: editorial@elifesciences.org.

Sample-size estimation

- You should state whether an appropriate sample size was computed when the study was being designed
- You should state the statistical method of sample size computation and any required assumptions
- If no explicit power analysis was used, you should describe how you decided what sample (replicate) size (number) to use

Please outline where this information can be found within the submission (e.g., sections or figure legends), or explain why this information doesn't apply to your submission:

A formal power analysis was not done: the main result reported here is not statistical in nature. The number of neurons recorded from is detailed in the first paragraph of Results, and all labeled neurons are summarized in the Supplementary Table, with cell numbers that reoccur in the figures so that the reader can identify specific cells. The number of recorded neurons is high for in vivo patch clamping in the brainstem, which are technically challenging recordings.

Replicates

- You should report how often each experiment was performed
- You should include a definition of biological versus technical replication
- The data obtained should be provided and sufficient information should be provided to indicate the number of independent biological and/or technical replicates
- If you encountered any outliers, you should describe how these were handled
- Criteria for exclusion/inclusion of data should be clearly stated
- High-throughput sequence data should be uploaded before submission, with a private link for reviewers provided (these are available from both GEO and ArrayExpress)

Please outline where this information can be found within the submission (e.g., sections or figure legends), or explain why this information doesn't apply to your submission:

Throughout the text a clear distinction is made between number of neurons (biological replicates) and number of repetitions within a neuron (technical replicates). The number of neurons recorded from and the number of neurons labeled are stated in the first paragraph of Results. For every population analysis it is indicated in how many neurons the respective analysis was done (Fig. 2B legend, Fig. 3, Fig. 4E, Fig. 5, Fig. 6, Fig. 7 Suppl. 1. legend) The number of repeated trials in acoustic responses is indicated (Fig. 5D Supplement 1 legend, Fig. 7 legend, Methods). As specified in Methods, one outlier was excluded in the calculation of mean +/- SEM of series resistance. Criteria for defining unlabeled neurons as LSO neurons are stated in the first paragraph of Results.

eLife Sciences Publications, Ltd is a limited liability non-profit non-stock corporation incorporated in the State of Delaware, USA, with company number 5030732, and is registered in the UK with company number FC030576 and branch number BR015634 at the address 1st Floor, 24 Hills Road, Cambridge CB2 1JP | August 2014

Statistical reporting

- Statistical analysis methods should be described and justified
- Raw data should be presented in figures whenever informative to do so (typically when N per group is less than 10)
- For each experiment, you should identify the statistical tests used, exact values of N, definitions of center, methods of multiple test correction, and dispersion and precision measures (e.g., mean, median, SD, SEM, confidence intervals; and, for the major substantive results, a measure of effect size (e.g., Pearson's r, Cohen's d)
- Report exact p-values wherever possible alongside the summary statistics and 95% confidence intervals. These should be reported for all key questions and not only when the p-value is less than 0.05.

Please outline where this information can be found within the submission (e.g., sections or figure legends), or explain why this information doesn't apply to your submission:

Statistical analysis methods are specified in Methods. Exact p-values are reported throughout the manuscript, as well as precision and dispersion values, the statistical test used, the value of the statistic with degrees of freedom, and measures of effect size when relevant.

(For large datasets, or papers with a very large number of statistical tests, you may upload a single table file with tests, Ns, etc., with reference to sections in the manuscript.)

Group allocation

- Indicate how samples were allocated into experimental groups (in the case of clinical studies, please specify allocation to treatment method); if randomization was used, please also state if restricted randomization was applied
- Indicate if masking was used during group allocation, data collection and/or data analysis

Please outline where this information can be found within the submission (e.g., sections or figure legends), or explain why this information doesn't apply to your submission:

Neurons were not allocated into experimental groups. As described in Methods, responses to different acoustic stimuli were attempted to be collected in all neurons, time permitting.

Additional data files ("source data")

- We encourage you to upload relevant additional data files, such as numerical data that are represented as a graph in a figure, or as a summary table
- Where provided, these should be in the most useful format, and they can be uploaded as "Source data" files linked to a main figure or table
- Include model definition files including the full list of parameters used
- Include code used for data analysis (e.g., R, MatLab)
- Avoid stating that data files are "available upon request"

Please indicate the figures or tables for which source data files have been provided:

MATLAB figures with embedded data will be made publicly available upon publication at figshare (https://figshare.com/).
